# Ketamine Induced Bladder Fibrosis Through MTDH/P38 MAPK/EMT Pathway

**DOI:** 10.3389/fphar.2021.743682

**Published:** 2022-01-28

**Authors:** Quan Zhu, Kaixuan Li, Haozhen Li, Feng Han, Zhengyan Tang, Zhao Wang

**Affiliations:** ^1^ Department of Urology, Xiangya Hospital, Central South University, Changsha, China; ^2^ National Clinical Research Center for Geriatric Disorders, Xiangya Hospital, Central South University, Changsha, China; ^3^ Provincial Laboratory for Diagnosis and Treatment of Genitourinary System Disease, Changsha, China

**Keywords:** Ketamine-induced cystitis, MTDH, EMT, p38 MAPK, bladder fibrosis

## Abstract

**Purpose:** Ketamine is an anesthetic in clinical, but it has also been used as an abusing drug due to its low price and hallucinogenic effects. It is proved that ketamine abusing would cause multiple system damage including the urinary system, which is called ketamine-induced cystitis (KIC). Bladder fibrosis is late stage in KIC and threaten abusers’ life. This study aimed to investigate the molecular mechanism of ketamine-induced bladder fibrosis.

**Methods:** Female Sprague Dawley (SD) rats were randomly divided into 3 groups. 2 groups were treated with tail vein injection of ketamine (25 mg/kg/day, 50 mg/kg/day ketamine hydrochloride solution, respectively) for 12 weeks, whereas the control group was treated with normal saline solution. In each group, rat bladders were extracted and samples were examined for pathological and morphological alterations via hematoxylin and eosin (HE) staining, Masson’s trichrome staining and immunohistochemistry (IHC). SV-HUC-1 cells were treated with different concentrations of ketamine solution (0, 0.1, 0.5, 1 mmol/L). Rat bladder and SV-HUC-1 cells were extracted protein and RNA for Western blot and RT-PCR detection. Metadherin (MTDH) siRNAs and overexpression plasmids were used to knock down and overexpress the relative genes. P38 mitogen-activated protein kinase (MAPK) inhibitor was utilized to inhibit the MAPK pathway.

**Results:** Rats in the ketamine group exhibited fibrosis compared to rats of the control group and fibrosis were also markedly upregulated in SV-HUC-1 cells after treated with ketamine, which were ketamine concentration-dependent. After treating with ketamine in SV-HUC-1 cells, there was an increase expression of MTDH, epithelial-mesenchymal transition (EMT) markers, P38 MAPK. MTDH knockdown would suppresses P38 MAPK/EMT pathway to inhibit fibrosis, however, MTDH overexpression could promote the pathway in SV-HUC-1 cells.

**Conclusion:** In rats and SV-HUC-1 cells ketamine-treated models, MTDH can regulate EMT through the P38 MAPK pathway to regulate the process of bladder fibrosis.

## Introduction

Ketamine, also known as K powder, is an N–methyl–D–aspartate receptor antagonist (Paulis et al., 2020). Ketamine was first used as an anesthetic in clinical after it was synthesized in 1962 (Dong et al., 2015; Wang et al., 2017). Because of its low price, short action time, and hallucinogenic effects, it has been used as a new type of drug-abusing in Hong Kong, Taiwan, and Europe in recent years (Yang et al., 2021; Tasi et al., 2009). After long-term abusing of ketamine, it will not only damage the nervous and cardiovascular system, but also damage the urinary system (Lee et al., 2017; Parkin et al., 2008). Scholars have reported that ketamine abusers are accompanied with severe lower urinary tract symptoms include frequency, dysuria, and hematuria, which was called ketamine-induced cystitis (KIC) clinically, furthermore bladder contracture were also detected in severe cases (Lee et al., 2017; Parkin et al., 2008). Scholars believe that direct toxicity by ketamine or its metabolites on the urothelial cells, activated the intrinsic apoptotic pathway, enhanced oxidative stress may be potential mechanisms (Chu et al., 2008; Gu et al., 2014; Liu et al., 2015). When KIC progresses to bladder fibrosis, or even bladder contracture, surgery isone of the key methods with a high complication rate and poor effects (Ng et al., 2013; Chu et al., 2008; Gu et al., 2014; Liu et al., 2015). Therefore, exploring the mechanism of ketamine-induced bladder fibrosis and delaying the progression of fibrosis is the vital therapy strategy of KIC and improve abusers’ life quality.

Metadherin (MTDH), which is also known as astrocyte upregulation factor −1 (AEG-1), was found in primary human fetal astrocytes after being treated with TNF-α or infected with HIV-1 (Britt et al., 2004; Brown and Ruoslahti, 2004). As a multifunctional protein, MTDH plays a role in processes including development, inflammation, and epithelial-mesenchymal transition (EMT) (Liu et al., 2016; Sarkar et al., 2013). Studies found that the MTDH expression was elevated in a variety of tumors. D, Pan et al. (2019) found that the viability, invasion, and migration of prostate cancer (Pca) cells is promoted after MTDH overexpression. Similar researches were also proved in non-small cell lung cancer, miR-145 and miR-497 inhibit EMT by targeting MTDH to inhibit metastasis and invasion (Yin et al., 2018).

The function of MTDH is gradually being recognized in fibrotic diseases. In the study of the unilateral ureteral occlusion (UUO) model, it was found that MTDH can promote renal fibrosis by regulating the EMT (Peng et al., 2019a). Previous studies have found that transforming growth factor-β can regulate ketamine-induced bladder fibrosis by regulating EMT (Wang et al., 2017). Is MTDH also involved in bladder fibrosis induced by ketamine? Does it play this role by regulating EMT? These are unclear and need to be further explored. As a stress-activated kinase, the P38 mitogen-activated protein kinase (MAPK) can be triggered by inflammation and growth factors (Kojonazarov et al., 2017). A large number of studies have shown that P38 MAPK is involved in the process of fibrosis. P38 MAPK was found to promote the transdifferentiation of fibroblasts into myofibroblasts in Graves’ ophthalmopathy (Hou et al., 2021), H G et al. found that CuSO4 treatment may induce pulmonary fibrosis through the activation of EMT induced by P38 MAPKs pathways (Guo et al., 2021).

The specific mechanism of bladder fibrosis after ketamine abuse is unknown. We hypothesize that MTDH is a key regulatory molecule, and it can regulate the process of bladder fibrosis by regulating EMT. In this study, the expression of MTDH and EMT marker proteins were detected in human bladder cell biochemistry Pillon (SV-HUC-1) cells and bladder tissue of rat models. Then, the expression of MTDH was regulated to study its regulatory effect. We provided evidence that the pro-fibrosis effect of MTDH is partly attributable to the promotion of the EMT by activating the P38 MAPK signaling pathway.

## Materials and Methods

### Animal and Ketamine Administration

All the experimental animals were performed on specific pathogen-free (SPF) female Sprague Dawley (SD) rats (Slack Jingda Experimental Animal corporation, Hunan, China) weighing between 200 and 250 g. A total of 24 SD rats were randomly distributed into three groups, eight rats per group, and received tail vein injection of normal saline solution (control group), tail vein injection of 25 mg/kg/day ketamine hydrochloride solution (2 ml/0.1 g, Fujian Gutian Pharmaceutical corporation) (low dose ketamine group), tail vein injection of 50 mg/kg/day ketamine hydrochloride solution (high dose ketamine group) for 12 weeks. The rats were weighed weekly to adjust the dosage of ketamine hydrochloride solution. Animals were raised at the Experimental Animal Center of Central South University (Hunan, China) and the animal experiments were approved by ethical review of experimental animal welfare in Central South University (NO: 2018sydw0225).

### Histological and Immunohistochemical Staining

The bladder tissue which was removed from the rats in the ketamine group and control group was fixed in 4% paraformaldehyde overnight, embedded in paraffin blocks, and sliced into a 4 μm section. Hematoxylin and eosin (H&E) and Masson trichrome staining (MTS) were performed to carry out the histopathologic evaluation such as the morphology and inflammatory cell infiltration of the bladder, the distribution of collagen. Immunohistochemical staining (IHC) was performed to evaluate the fibrosis, MTDH expression, EMT markers of the bladder in ketamine group rats compared with the control group. Briefly, embedded bladder tissue was first deparaffinized and then subjected to a microwave oven for antigen retrieval in citrate buffer. The sections were incubated overnight at 4°C in rabbit MTDH antibody (1:2000, ab227981, Abcam, Cambridge, United Kingdom), mouse E-cadherin (1: 200, ab76055, Abcam, Cambridge, United Kingdom), mouse vimentin (1: 500, ab8069, Abcam, Cambridge, United Kingdom), rabbit fibronectin antibody (1:500, ab2413, Abcam, Cambridge, United Kingdom), and rabbit collagen I antibody (1:500, ab138492, Abcam, Cambridge, United Kingdom) after being blocked with 3% H2O2. And then the sections were incubated with biotinylated secondary antibody (1:200; CWBio, Beijing, China) for 30 min at room temperature, and development was achieved with 3,3′-diaminobenzidine.

### Cell Culture and Treatment

The human bladder cell biochemistry Pillon (SV-HUC-1) was obtained from Guangzhou Cellcook Biotech Co., Ltd. (Cellcook, cc4009, China) and cultured in F-12K medium (Gibco, NY, United States) containing 10% fetal bovine serum (FBS, Gibco, NY, United States) at 37°C constant temperature and 5% CO2. SV-HUV-1 was stimulated with ketamine solution (0, 0.1, 0.5, 1 mmol/L) diluted by medium for 48 h. The SB203580 (10 μmmol/L, P38 MAPK selective inhibitor) was treated with SV-HUC-1 in the corresponding group.

### Real-Time Quantitative RT-PCR

Total RNA was extracted from SV-HUV-1 cell lines using RNAiso Plus reagent (Takara Bio Inc., Otsu, Shiga, Japan) and cDNA was synthesized using PrimeScript™ RT reagent kit (Takara Bio Inc. Otsu, Shiga, Japan). The primers designed details were shown in Supplementary S1. The two-step real-time RT-PCR was performed using SYBR Green Reagent (United States EVERBRIGHT^®^ INC., Suzhou, China) and C1000 Touch Thermal Cycler, CFX96 Real-Time System (Bio-Rad) according to manufacturer’s instructions. The results were calculated by the 2−ΔΔCt method and data are expressed as a ratio of the control gene GAPDH.

### Western Blot

The SV-HUV-1 cells and bladder tissues were lysed with RIPA (Thermo, United States) containing protease cocktail (Servicebio, Wuhan, China) to obtain the total protein. The protein was separated and transferred via cataphoresis using 10% SDS-PAGE gel electrophoresis and PVDF membranes. The primary antibodies against rabbit MTDH antibody (1:1,000, ab227981, Abcam, Cambridge, United Kingdom), mouse E-cadherin (1: 1,000, ab76055, Abcam, Cambridge, United Kingdom), mouse vimentin (1: 1,000, ab8069, Abcam, Cambridge, United Kingdom), rabbit P38 (1:1,000, #8690, Cell Signaling Technology, United States), rabbit P-P38 (1:1,000, #4511, Cell Signaling Technology, United States), rabbit fibronectin (1:500, ab2413, Abcam, Cambridge, United Kingdom), rabbit collagen Ⅰ (1:1,000, ab260043, Abcam, Cambridge, United Kingdom), rabbit α-SMA (1:1,000, ab5694, Abcam, Cambridge, United Kingdom), mouse Flag-Tag (1:2000, T0003, Affinity, Australia) and GAPDH (1:3,000, ab8245, Abcam, Cambridge, United Kingdom) were incubated with the membranes overnight at 4°C. After incubating, blots were detected by secondary antibody and visualized by the ECL assay (Milliporesigma) and the bands were analyzed with Image J^®^ software.

### Cell Transfected

SV-HUC-1 was seeded in a 6-well cell culture cluster. MTDH siRNA and MTDH overexpression plasmid were bought from Sangon Biotech (Shanghai, China) and the details of the sequence are in Supplementary S1. Lipofectamine 3,000 (Invitrogen, United States) was diluted with a serum-free neurobasal medium and mixed with RNA. The SV-HUC-1 cells were treated with the solution for 12 h.

### CCK-8 Assay

SV-HUC-1 cells were inoculated into 96-well plates (1 × 104 cells/well) for 24 h, and then stimulated with ketamine solution (0.1–2 mmol/L) diluted by medium for 48 h. After ketamine treatment, CCK-8 solution (NCM, Suzhou, China) was added to the 96-well (10 ul/well) and incubated with SV-HUC-1 for 2 h, the absorbance at 450 nm was detected via a microplate reader (Molecular Device, California, United States).

### Urination Spot Test

The urination spot test spot test was performed in a metabolic cage. The test paper used for urine spots was a customized test paper that turns red when exposed to urine, which could accurately indicate the liquid infiltration. Twelve weeks after the injection of ketamine and 0.9% saline, the rats in the experimental group and the control group were moved into a metabolic cage to collect urine. The urine spot test was carried out from 8 am to 12 o’clock in the morning.

### Urodynamic Test

After anesthesia, the rats were fixed in the supine position, and 75% ethanol was used to disinfect the skin of the abdomen and perineum. After connecting each end of the cystometry tube and the three-way tube and venting the air, they were inserted into the bladder along the urethra of the rat. Gently press the rat’s abdomen to empty the bladder of residual urine. After the computer point signal was reset to zero, the micro-injection pump was turned on, and the normal saline preheated to 37°C was continuously perfused at a rate of 6 ml/h. Observe the urinary interval by observing the fluid outflow interval of the external urethra; by observing the number of times the water pressure is higher than the baseline 10 cm water column within 3 min before the leakage of urine to obtain the frequency of unstable contraction of the bladder detrusor.

### Statistical Analyses

SPSS 20.0 (IBM, New York, United States) was utilized to perform the statistical analyses. Data were shown as mean ± standard error of the mean (SEM) according to the results of three independent repeated experiments. Two-way analysis of variance (ANOVA) was used to analyze the differences. *p* < 0.05 or less was considered to be statistically significant.

## Result

### Ketamine Induced Fibrotic Changes in Rats Bladder and SV-HUC-1 Cells

**FIGURE 1:**
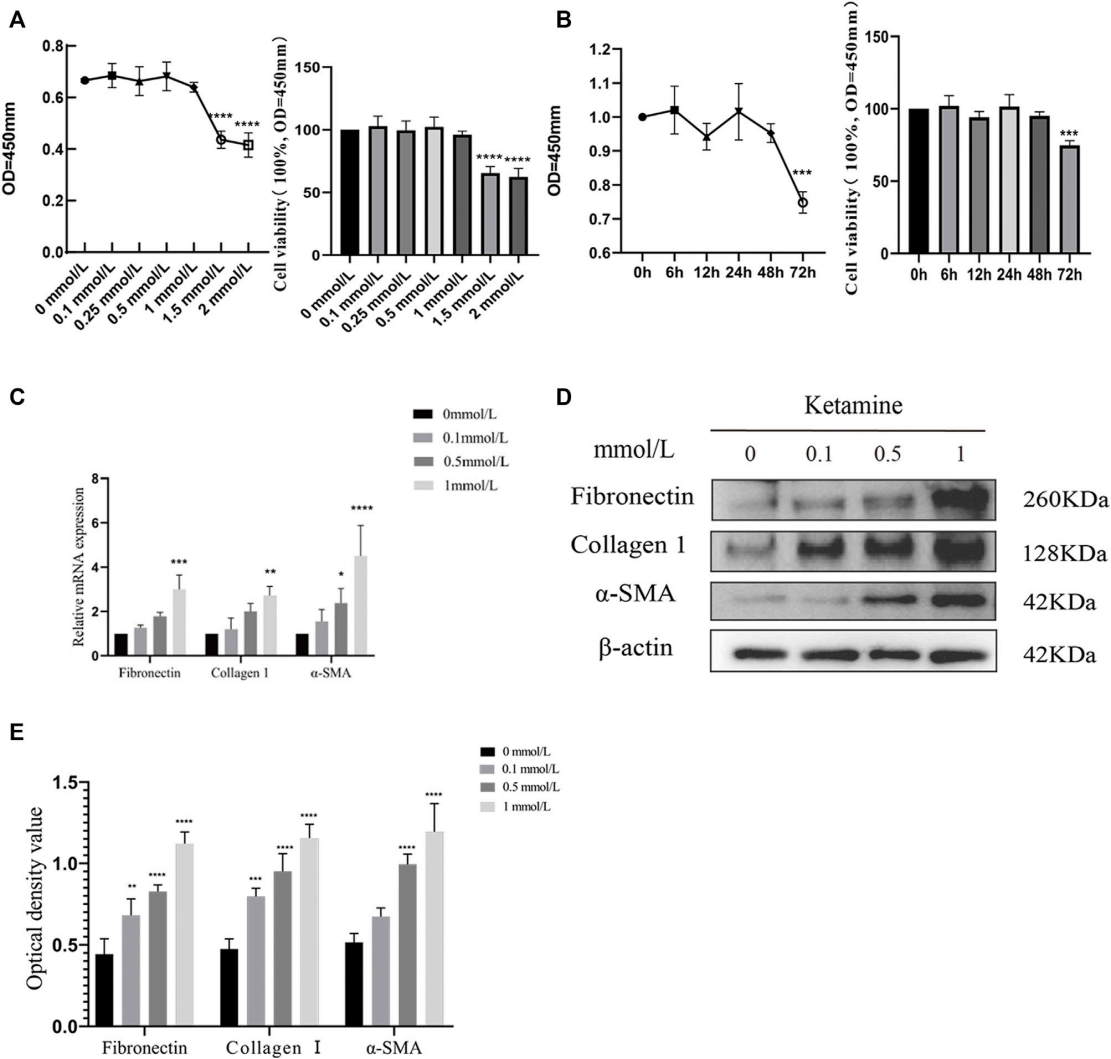
Ketamine causes fibrotic changes in SV-HUC-1 cells. **(A)** The cytotoxicity of 48 h treated ketamine was tested by a CCK8 kit. When the concentration of ketamine reaches 1.5 mmol/L, cell viability decreases significantly,****p < 0.0001 compared with 0 mmol/L. **(B)** The cytotoxicity of ketamine was tested by a CCK8 kit in a time-dependent manner. When the time of ketamine treatment reaches 72 h, cell viability decreases significantly, ***p < 0.001 compared with time less than 48 h. **(C)** Relative RNA levels as determined by the RT-PCR (treated for 48 h). **(D)** Representative bands from Western blot analyses of the levels of the fibronectin, collagen Ⅰ, α-SMA protein in SV-HUC-1 cells after being treated with different concentrations of ketamine (treated for 48 h). **(E)** Relative levels of the fibronectin, collagen Ⅰ, α-SMA protein compared to β-actin. *p < 0.05 compared with 0 mmol/L; **p < 0.05 compared with 0 mmol/L; ***p < 0.001 compared with 0 mmol/L; ****p < 0.0001 compared with 0 mmol/L. N=3.

**FIGURE 2:**
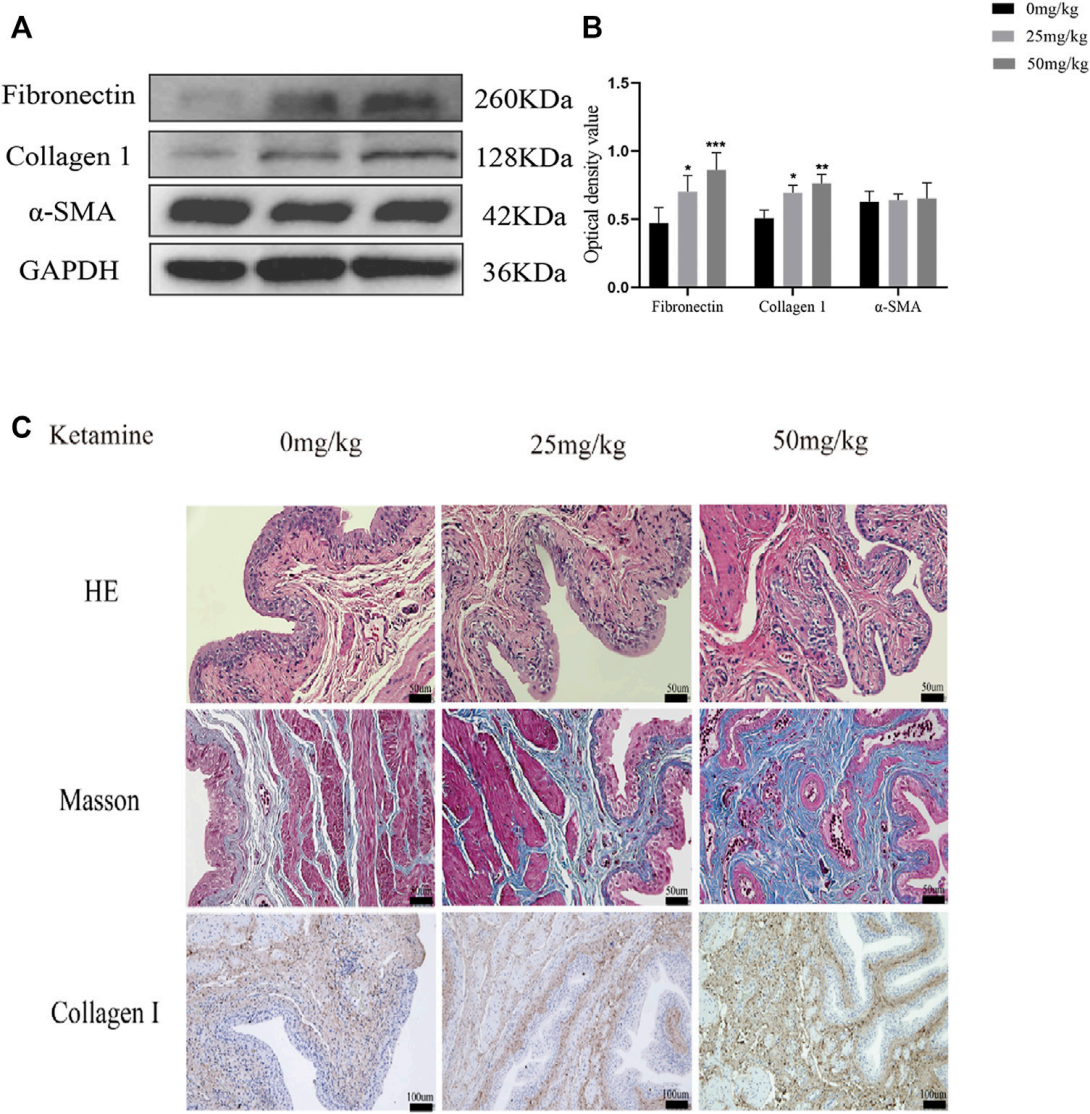
Ketamine induced fibrotic changes in rat’s bladder. **(A)** Representative bands from Western blot analyses of the levels of the fibronectin, collagen Ⅰ and α-SMA proteins in rat bladder tissue after being treated with different concentrations of ketamine (treated for 12 w). **(B)** Relative levels of the fibronectin and collagen Ⅰ proteins compared to GAPDH. *P < 0.05 compared with 0 mg/kg, **p < 0.01 compared with 0 mg/kg; ***p < 0.001 compared with 0 mg/kg; ****p < 0.0001 compared with 0 mg/kg. **(C)** Representative micrographs of HE staining and Masson’s trichrome staining and IHC staining for collagen Ⅰ in rat bladder tissue. Scale bar in HE staining and Masson’s trichrome staining is 50 μm; in IHC for collagen Ⅰ staining is 100 μm (treated for 12 w). N=3.

We determined the effects of different concentrations of ketamine (0, 0.1, 0.5, 1, 1.5, and 2.0 mmol/L) on SV-HUC-1 cells using a CCK-8 kit to evaluate the cytotoxicity of ketamine *in vitro*. After 48 h of treatment, the viability of SV-HUC-1 cells cultured with 1.5 mmol/L ketamine was significantly decreased (Figure 1A), indicating that ketamine does not induce significant cell toxicity at concentrations less than 1 mmol/L in SV-HUC-1 cells. Ketamine was used at a concentration of 0, 0.1, 0.5, 1 mmol/L in subsequent experiments to investigate the effect of ketamine on SV-HUC-1 cells. Then we also analyzed the time-dependent cytotoxicity of 1 mmol/L ketamine and found that 48 h treatment was appropriate (Figure 1B). With the concentration of ketamine treatment increased, fibronectin, collagen Ⅰ, and α-SMA protein and RNA expression levels were upregulated (Figures 1C,D,E). And we analyzed the expression of MTDH, EMT markers and fibrotic markers in SV-HUC-1 cells after treatment with 1 mmol/L ketamine in a time-dependent (0, 6, 12, 24, 48, 72 h) manner (Supplementary Figures S2A,B), the result show that as time increases, MTDH, vimentin and fibrosis indicators increase with time, and E-cadherein decreasesd with time. In the low dose ketamine group, 1 rat died after ketamine injection, in the high dose ketamine group, 1 died during the injection, and 1 died after the injection. The cause of death was considered to be the inhibitory effect of ketamine on respiration. Fibronectin, collagen Ⅰ were also elevated in the rat bladder after ketamine treatment (Figures 2A,B). Compared with the control group, it can be observed in the experimental group exfoliation and thinning of the bladder mucosa and collagen fiber deposition in bladder tissue by HE staining and Masson’s trichrome staining, and the positive expression of collagen Ⅰ was elevated in IHC staining (Figure 2C). And we analyzed the expression of MTDH, EMT markers and fibrotic markers in Rat after treatment with 25 mg/kg ketamine in a time-dependent (0, 4, 8, 12 w) manner (Supplementary Figures S2A,B). In addition, the evaluation of bladder function (urodynamics and urination spot test) showed that the bladder function damaged after treated with ketamine. According to the urination spot test, the number of urination in the control group was 3.23 ± 1.23, and the number of urination in the low and high dose ketamine group were 6.65 ± 1.97 and 8.76 ± 2.45times (*p* = 0.01). And the urodynamics test showed that in the ketamine-induced cystitis rat model group, the frequency of urination increased, the interval between urination was shortened, and the unstable contraction increased (Supplementary Figures S1A,B; Supplementary Table S2). The above results suggest that ketamine causes increased fibrosis markers in the SV-HUC-1 cells and rat model.

### Ketamine Promotes EMT, and MTDH Might be a Novel Biomarkers in Bladder Fibrosis

**FIGURE 3:**
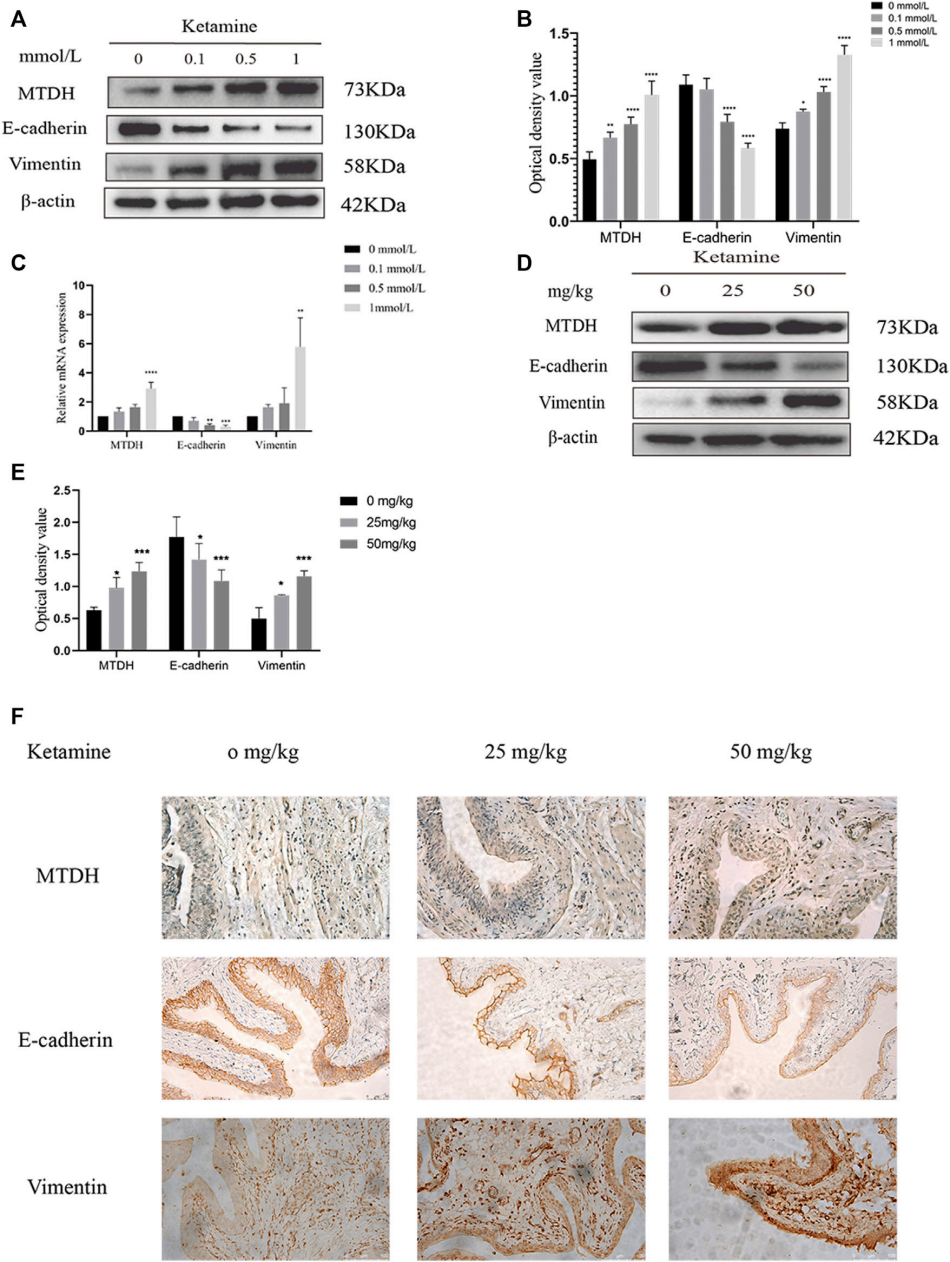
Expression of MTDH and EMT markers in SV-HUC-1 cells and rat bladder after treatment with different concentrations of ketamine. **(A)** Representative bands from Western blot analyses of the levels of the MTDH, E-cadherin, vimentin protein in SV-HUC-1 cells after being treated with different concentrations of ketamine (treated for 48 h). **(B)** Relative levels of the MTDH, E-cadherin, vimentin protein compared to GAPDH in SV-HUC-1 cells. *p < 0.05 compared with 0 mmol/L; **p < 0.01 compared with 0 mmol/L; ****p < 0.0001 compared with 0 mmol/L. **(C)** Relative levels of the MTDH, E-cadherin, vimentin mRNA compared to GAPDH. **p < 0.01 compared with 0 mmol/L, ***P < 0.001 compared with 0 mmol/L, ****p < 0.0001 compared with 0 mmol/L (treated for 48 h). **(D)** Representative bands from Western blot analyses of the levels of the MTDH, E-cadherin, vimentin protein in rat bladder tissue after being treated with different concentrations of ketamine (treated for 12 w). **(E)** Relative levels of the MTDH, E-cadherin, vimentin protein compared to GAPDH in rat bladder tissue. *p < 0.05 compared with 0 mg/kg; ***p < 0.0001 compared with 0 mg/kg. **(F)** Representative micrographs of IHC staining for MTDH, E-cadherin, vimentin in rat bladder tissue (treated for 48 h). The scale bar is 50 μm. N=3.

To elucidate the underlying mechanism of ketamine-induced fibrosis, MTDH and EMT markers expression were investigated. Compared with the control group, the protein and RNA expression of MTDH and vimentin were increased, and the expression of E-cadherin was decreased in SV-HUC-1 cells after ketamine treatment (Figures 3A–C). Similar results can be seen in rat bladder tissues, the expression of MTDH and vimentin protein increased and the expression of E-cadherin was decreased in the experimental group (Figures 3D,E). In IHC staining, the positive expression of MTDH and vimentin increases, and the positive expression of E-cadherin decreases in rat bladder after ketamine treatment (Figure 3F). These results suggest that ketamine can promote the initiation of EMT, and MTDH may be one of the important biomakers of ketamine induced bladder fibrosis.

### MTDH Knockdown Suppresses EMT and Fibrosis, and MTDH Overexpression Promotes the Process

**FIGURE 4:**
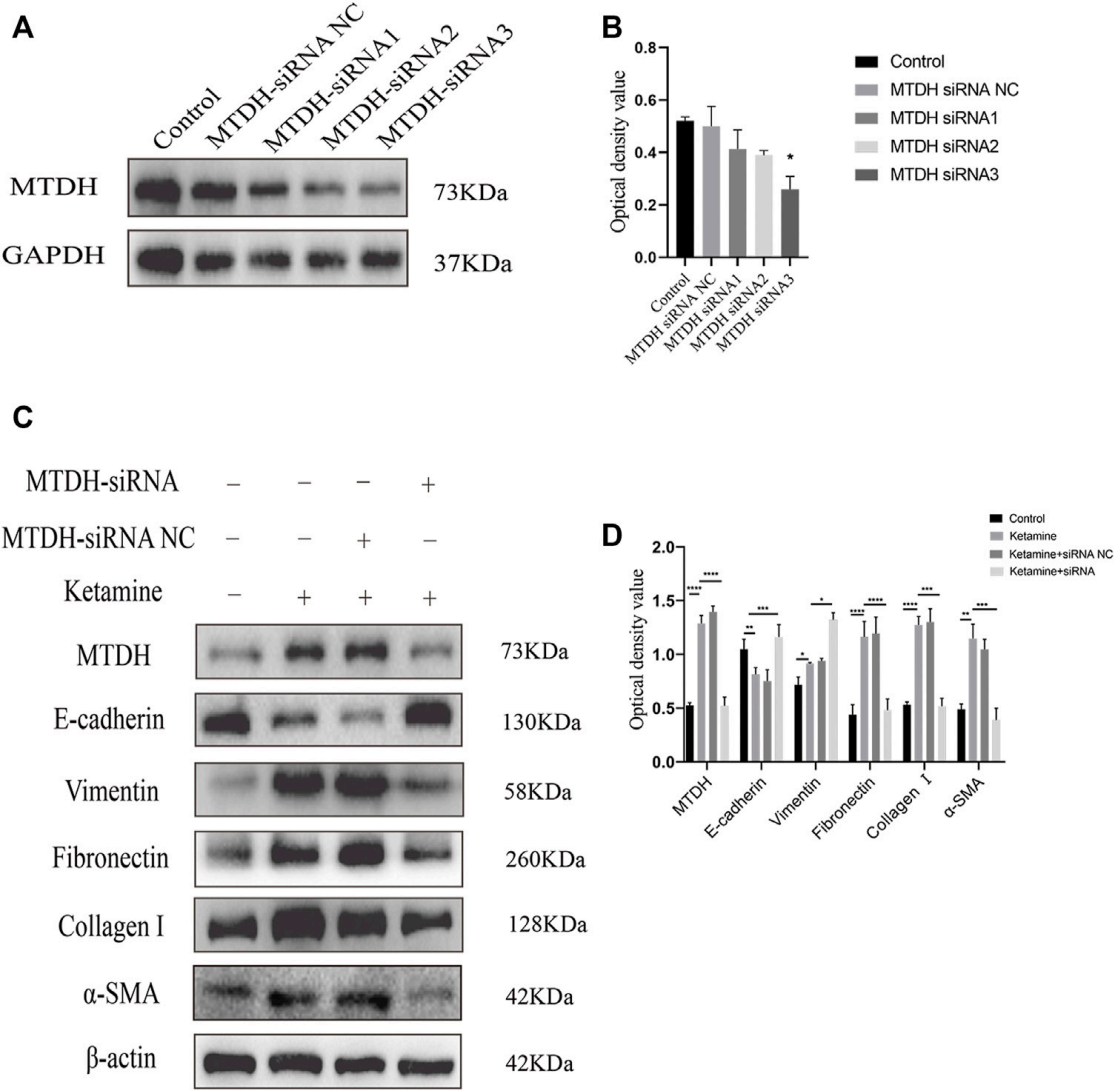
Selection of MTDH siRNA and the expression of EMT and fibrosis marker proteins after silencing MTDH. **(A)** Representative bands from Western blot analyses of the levels of the MTDH in SV-HUC-1 cells after being transfected with MTDH siRNA negative control (NC), MTDH siRNA 1, MTDH siRNA 2, MTDH siRNA 3. **(B)** Relative levels of the MTDH compared to GAPDH. *p < 0.05 compared with control. **(C)** Representative bands from Western blot analyses of the levels of the MTDH, E-cadherin, vimentin, fibronectin, collagen Ⅰ, α-SMA protein in SV-HUC-1 cells after being treated with ketamine, ketamine and MTDH siRNA NC, ketamine and MTDH siRNA 3 (treated for 48 h). **(D)** Relative levels of the MTDH, E-cadherin, vimentin, fibronectin, collagen Ⅰ, α-SMA protein compared to β-actin. *p < 0.05; **p < 0.01; ***p < 0.0001; ; ****p < 0.0001. N=3.

**FIGURE 5:**
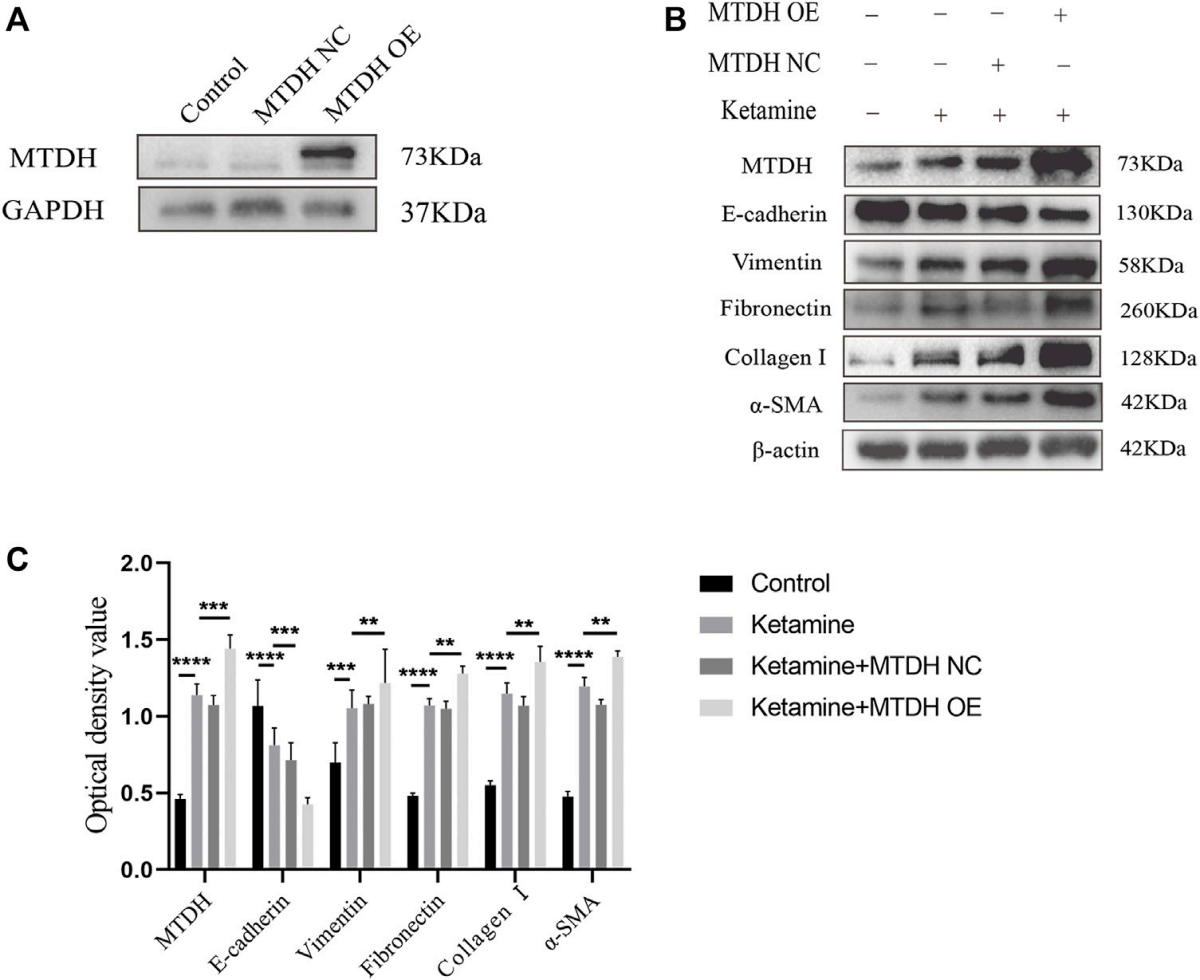
MTDH, EMT, and fibrosis markers protein expression after MTDH overexpression. **(A)** Representative bands from Western blot analyses of the levels of the MTDH in SV-HUC-1 cells after being transfected with MTDH overexpression NC plasmid and MTDH overexpression plasmid. **(B)** Representative bands from Western blot analyses of the levels of the MTDH, E-cadherin, vimentin, fibronectin, collagen Ⅰ, α-SMA protein in SV-HUC-1 cells after being treated with ketamine, ketamine and MTDH overexpression NC plasmid, ketamine and MTDH overexpression plasmid (treated for 48 h). **(C)** Relative levels of the MTDH, E-cadherin, vimentin, fibronectin, collagen Ⅰ, α-SMA protein compared to β-actin. **p < 0.01; ***p < 0.0001; ****p < 0.0001. N=3.

We further investigated the role of MTDH in ketamine-induced bladder fibrosis by MTDH knockdown or overexpression. Successful MTDH knockdown was confirmed by WB analysis. MTDH expression in SV-HUC-1 cells was shown an obvious decrease following the knockdown (Figures 4A,B). Small interfering RNAs (siRNAs) three were chosen to perform subsequent experiments for the best silence efficiency. In addition, inhibited MTDH expression was shown to suppress the expression of vimentin and promote the expression of E-cadherin, also promoted the expression of fibrosis markers fibronectin, collagen Ⅰ, and α-SMA in SV-HUC-1 cells (Figures 4C,D). Following the overexpression of MTDH in SV-HUC-1 cells, the expression of exogenous MTDH had been confirmed (Figure 5A). Conversely, MTDH overexpression led to an increase of vimentin and a decrease of E-cadherin (Figures 5B,C), and increase the expression of fibrosis markers fibronectin, collagen Ⅰ, and α-SMA in SV-HUC-1 cells. MTDH-overexpression only group and MTDH-siRNA inhibitor only group were showed in Supplementary Figures S2C–F. The above results suggest that MTDH could promote the occurrence of bladder fibrosis by regulating EMT.

### MTDH Regulates Bladder Fibrosis Partially Through the P38 MAPK/EMT Pathway

**FIGURE 6:**
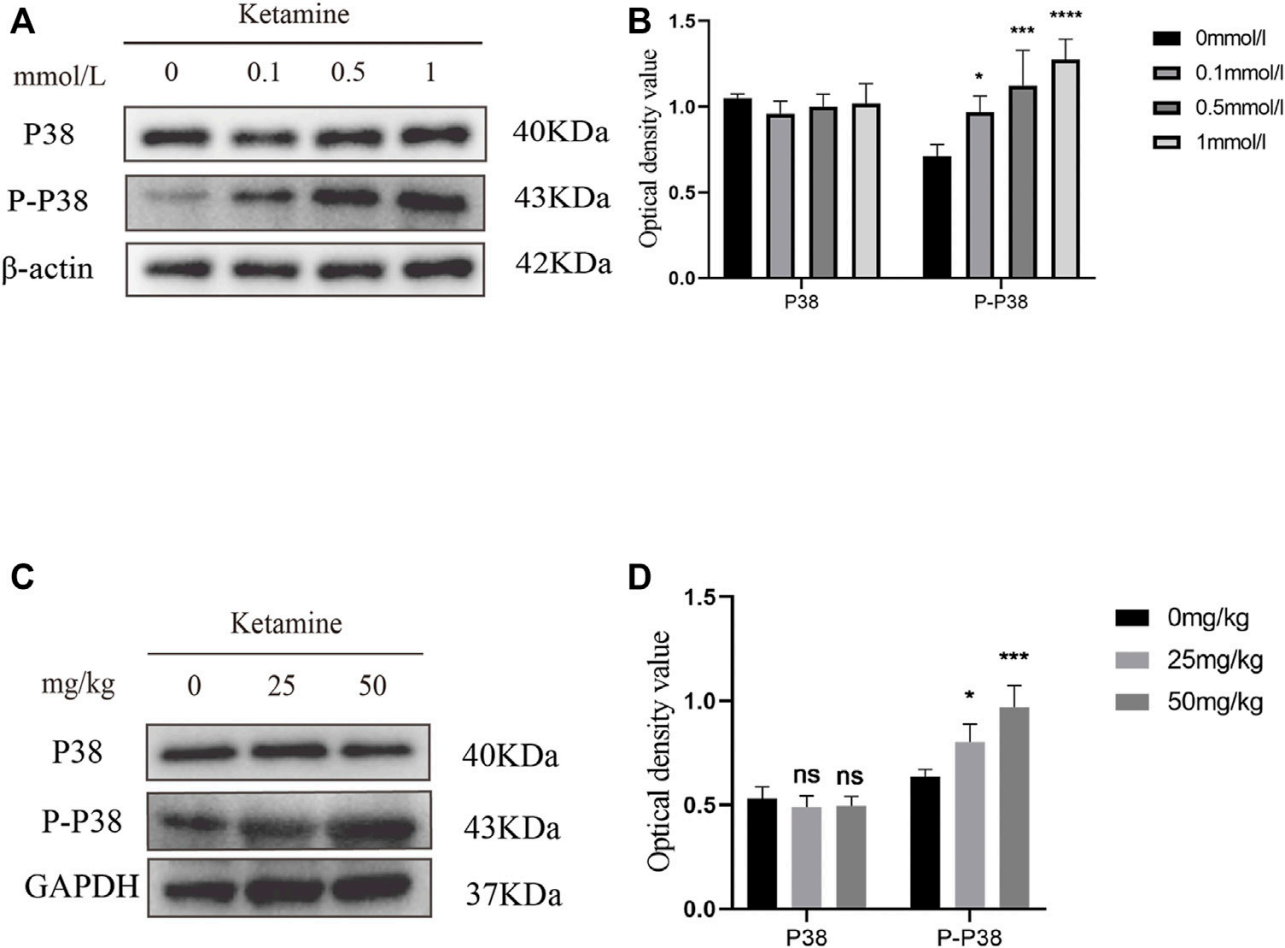
The expression of P38 and P-P38 protein in SV-HUC-1 cells and rat bladder after treatment with different concentration of ketamine. **(A)** Representative bands from Western blot analyses of the levels of the P38 and P-P38 protein in SV-HUC-1 cells after treatment with different concentrations of ketamine (treated for 48 h). **(B)** Relative levels of the P38 and P-P38 protein compared to β-actin in SV-HUC-1 cells. *p < 0.05 compared with 0 mmol/L group, ***p < 0.0001 compared with 0 mmol/L group, ****p < 0.0001 compared with 0 mmol/L group. **(C)** Representative bands from Western blot analyses of the levels of the P38 and P-P38 protein in rat bladder after treatment with different concentration of ketamine (treated for 12 w). **(D)** Relative levels of the P38 and P-P38 protein compared to β-actin in rat bladder. *p < 0.05 compared with 0 mg/kg group, ***p < 0.0001 compared with 0 mg/kg group. N=3.

**FIGURE 7:**
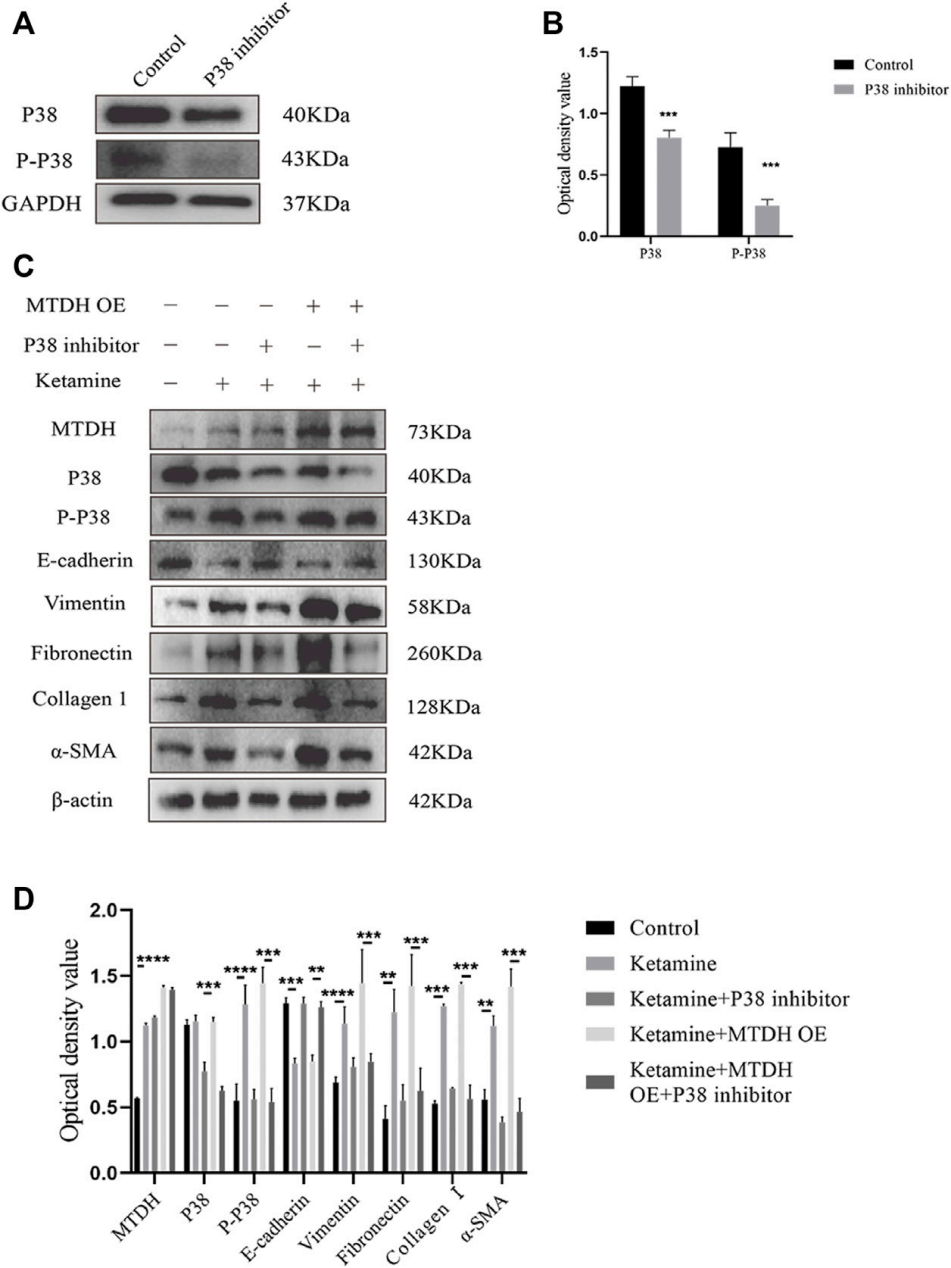
The influence of P38 inhibitor SB203580 on the regulation of MTDH in SV-HUC-1 cells. **(A)** Representative bands from Western blot analyses of the levels of the P38 and P-P38 protein in SV-HUC-1 cells after treatment with SB203580. **(B)** Relative levels of the P38 and P-P38 protein compared to GAPDH. ***p < 0.001 compared with control group, **p < 0.01 compared with control group. **(C)** Representative bands from Western blot analyses of the levels of the MTDH, P38, and P-P38, E-cadherin, vimentin, fibronectin, collagen Ⅰ, α-SMA protein in SV-HUC-1 cells after treatment with ketamine, ketamine and SB203580, ketamine and MTDH overexpression plasmid, ketamine and MTDH overexpression plasmid and SB203580 (treated for 48 h). **(D)** Relative levels of the MTDH, P38, and P-P38, E-cadherin, vimentin, fibronectin, collagen Ⅰ, α-SMA protein compared to β-actin. **p < 0.01; ***p < 0.001; ****p < 0.0001. N=3.

To investigate whether MTDH regulates EMT through the P38 MAPK pathway to promote bladder fibrosis, we measured the expression of P38 and phosphorylated (P)-P38 protein in SV-HUC-1 and rat bladder after ketamine treatment. In comparison with the control group, the expression levels of P-P38 protein were shown to be significantly increased in the ketamine treatment group, while there was no significant changes in the expression of P38 protein (Figures 6A–D). When P38 MAPK inhibitor SB203580 was added to SV-HUC-1 cells, the expression of P38 and P-P38 protein were inhibited to varying degrees (Figures 7A,B). Ketamine, ketamine and SB203580, ketamine and MTDH overexpression plasmid, ketamine and MTDH overexpression plasmid and SB203580 were added respectively in SV-HUC-1 cells. Compared with the ketamine group, the promotion of ketamine on EMT and fibrosis was significantly inhibited, but the expression of MTDH did not change significantly after adding SB203580. Compared with the addition of ketamine and MTDH overexpression plasmid group, the promotion of MTDH on EMT and fibrosis was significantly inhibited, while the expression of MTDH did not change significantly after adding SB203580 (Figures 7C,D). P38 inhibitor only group was showed in Supplementary Figures S2G,H. The results suggest that MTDH may promote bladder fibrosis partially through the P38 MAPK/EMT pathway.

## Discussion

In the present study, underlying mechanisms of ketamine-induced bladder fibrosis were studied using SD rats and SV-HUC-1 cells. Our results suggested that EMT is an important process of bladder fibrosis. Furthermore, we also found that a novel pro-fibrotic molecule MTDH can regulate EMT through the P38 MAPK signaling pathway involved in bladder fibrosis induced by ketamine. MTDH might be a potential biomarkers in detection ketamine induced bladder fibrosis.

Since it was first reported in 2007, a large number of the literature revealed that long-term use of ketamine can cause damage to the urinary system (Shahani et al., 2007; Kalsi et al., 2011; Winstock et al., 2012). Decreased bladder capacity and unstable detrusor contraction were found. However, bladder contracture were also reported in severe patients, some patients gradually developed upper urinary tract damage such as hydronephrosis and renal dysfunction (Chen et al., 2020). Bladder fibrosis is a late damage stage in the progression of KIC. Once it occurs, it might indicate a potential poor prognosis for the patient. Song, M. et al. team established a rat model of bladder fibrosis by injecting ketamine through the tail vein for 2 weeks at a dose of 25 mg/kg (Kim et al., 2016). Causing irreversible fibrosis of the bladder usually requires a long-term abuse process. So, in our research, we extended the time of tail vein injection to 12 weeks to better meet the clinical characteristics. According to the results of CCK8, there is no significant decrease in cell viability before the ketamine concentration is less than 1.5 mmol/L, so we choose the ketamine treatment concentration to be 0, 0.1, 0.5, 1 mmol/L we verified the establishment of a model of bladder fibrosis induced by ketamine in SV-HUC-1 cells and SD rats by assessing changes in the expression of fibrosis markers fibronectin, collagen Ⅰ, α-SMA, laying a solid foundation for subsequent research.

EMT is involved in a variety of biological processes, such as tissue repair and pathology, including cancer and cataract (Sisto et al., 2021). It also plays an important role in the progression of fibrotic diseases. EMT is characterized by loss of epithelial cell-specific proteins, such as E-cadherin, and increased expression of mesenchymal markers, including vimentin, α-SMA (Lamouille et al., 2014). In our study, after being treated with ketamine in SD rats and SV-HUC-1 cells, the expression of the epithelial marker E-cadherin decreased, while the expression of the mesenchymal marker vimentin increased, suggesting that ketamine can induce the transition of bladder epithelial cells to mesenchymal cells. Studies have shown that epithelial cells can transform into myofibroblasts through EMT to synthesize extracellular matrices such as collagen Ⅰ and fibronectin (Iwano et al., 2002; Li et al., 2016). Our research also found that after ketamine treatment, the expression of collagen Ⅰ and fibronectin increased. Prolonged activation of the EMT process may cause inflammation, and inflammation is also an effective induction of EMT, thus indicating that the two phenomena can maintain each other (Zeisberg et al., 2003; Sisto et al., 2018). The above results suggest that after ketamine treatment, bladder epithelial cells may partly transform into myofibroblasts, gaining the ability to synthesize extracellular matrix, and ultimately cause bladder fibrosis.

Most of the current research on MTDH focuses on its role in tumors. Previous studies have shown that MTDH can activate several classic cancer-promoting signaling pathways, such as EMT, NF- κ B and MAPK to enhance tumor progression and metastasis (Wang et al., 2013; Chang et al., 2016; El-Ashmawy et al., 2019). It has been reported that MTDH may participate in the process of renal fibrosis by regulating the EMT process (Peng et al., 2019a; Peng et al., 2019b). The expression of MTDH in bladder tissue and SV-HUC-1 increased significantly after ketamine treatment. It suggests that MTDH may be involved in the process of bladder fibrosis. After knocking down MTDH, we found that the process of EMT and bladder fibrosis were also inhibited, and when MTDH expression unregulated, the result was reversed. It was suggested that MTDH might be a key molecule in ketamine-induced bladder fibrosis, and it can play its role in promoting fibrosis by regulating EMT.

Studies have found that P38 MAPK is closely related to myocardial fibrosis, pulmonary fibrosis, and liver fibrosis (Gu et al., 2016; Dong et al., 2020; Goda et al., 2020). Pirfenidone, as one non-selective P38 inhibitor, has been approved for clinical treatment of idiopathic pulmonary fibrosis (Valeyre et al., 2014). To verified whether the P38 MAPK pathway is activated in ketamine-induced bladder fibrosis, the expression of P38 and P-P38 proteins was assessed in rats and SV-HUC-1 cells models in our research. The ratio of P-P38 to total P38 increased compared with the control group, prompt that the P38 MAPK pathway is activated after ketamine treatment. After knockdown and overexpression of MTDH, the ratio of P-P38 to P38 also decreased or increased, respectively. When P38 inhibitor was added, it can partially block the regulation of EMT by MTDH, thereby alleviating its effect on bladder fibrosis. The above results indicate that the expression of P38 MAPK is partially regulated by MTDH, and the function of MTDH regulating EMT is partially through P38 MAPK.

In this study, we demonstrated that ketamine induced bladder fibrosis involves pathway MTDH/P38 MAPK/EMT. But the study has limitations as follows (I) Clinical investigation and data were not included in the present study. And the expression level of MTDH/P38 MAPK/EMT pathway markers were not detected in clinical bladder samples and this awaits further studies (II) We mainly verified the MTDH/P38 MAPK/EMT pathway *in vitro*, but *in vivo*, we did not study the mechanism of MTDH as a key molecule of ketamine induced bladder fibrosis and its possible target for the treatment. However, we revealed that MTDH might be a vital biomarker and treatment target of ketamine induced bladder fibrosis.

## Conclusion

In conclusion, we revealed that in rats and SV-HUC-1 cells models, MTDH can regulate EMT through the P38 MAPK pathway to partly regulate the process of bladder fibrosis. MTDH may serve as a novel biomarker and therapeutic target in bladder fibrosis induced by ketamine abuse.

## Data Availability

The original contributions presented in the study are included in the article/Supplementary Material, further inquiries can be directed to the corresponding author.
