# Supplementary material for: Ketamine Induced Bladder Fibrosis Through MTDH/P38 MAPK/EMT Pathway
Source: Front Pharmacol. 2022 Jan 28;12:743682. doi: 10.3389/fphar.2021.743682 (PMC8837385; doi:10.3389/fphar.2021.743682)
Supplement: Supplementary file 1 [file DataSheet1.docx]

Supplementary Material

**Table S1**. Overview of sequence used for RT-PCR and MTDH knockdown and overexpression

| Targets | Sequence |
| --- | --- |
| MTDH-Human | F: CCAGTTTCTCAGTCTACCACTT  R: CCCAGACCATTCATCATCGATA |
| E-CADHERIN-Human | F: CTCAGAAGACAGAAACGAGACT  R: AACCAGGTTCTTTGGAAATTCG |
| VIMENTIN-Human | F: TGAATGACCGCTTCGCCAACTAC  R: CTCCCGCATCTCCTCCTCGTAG |
| FIBRONECTIN-Human | F: AATAGATGCAACGATCAGGACA  R: GCAGGTTTCCTCGATTATCCTT |
| COLLAGEN I-Human | F: AAAGATGGACTCAACGGTCTC  R: CATCGTGAGCCTTCTCTTGAG |
| GAPDH-Human | F: GTCTCCTCTGACTTCAACAGCG  R: ACCACCCTGTTGCTGTAGCCAA |
| MTDH-siRNA1 | F: CGUGAUAAGGUGCUGACUGAUTT  R: AUCAGUCAGCACCUUAUCACGTT |
| MTDH-siRNA2 | F: AUCAGUCAGCACCUUAUCACGTT  R: GAUAUAGGGUUGAUUACGGCUTT |
| MTHD-siRNA3 | F: CCAAUACUACAAGAGACAGAUTT  R: AUCUGUCUCUUGUAGUAUUGGTT |
| MTDH-OE plasmid | CTGGTTTAGTGAACCGTCAGATCCGCTAGTAATACGACTCACTATAGGGAGAGGATCCGGTACCGAGGAGATCTGCCGCCGCGATCGCCATGGCTGCACGGAGCTGGCAGGACGAGCTGGCCCAGCAGGCCGAGGAGGGCTCGGCCCGGCTGCGGGAAATGCTCTCGGTCGGCCTAGGCTTTCTGCGCACCGAGCTGGGCCTCGACCTGGGGCTGGAGCCGAAACGGTACCCCGGCTGGGTGATCCTGGTGGGCACTGGCGCGCTCGGGCTGCTGCTGCTGTTTCTGCTGGGCTACGGCTGGGCCGCGGCTTGCGCCGGCGCCCGCAAAAAGCGGAGGAGCCCGCCCCGCAAGCGGGAGGAGGCGGCGGCCGTGCCGGCCGCGGCCCCCGACGACCTGGCCTTGCTGAAGAATCTCCGGAGCGAGGAACAGAAGAAGAAGAACCGGAAGAAACTGTCCGAGAAGCCCAAACCAAATGGGCGGACTGTTGAAGTGGCTGAGGGTGAAGCTGTTCGAACACCTCAAAGTGTAACAGCAAAGCAGCCACCAGAGATTGACAAGAAAAATGAAAAGTCAAAGAAAAATAAGAAGAAATCAAAGTCAGATGCTAAAGCAGTGCAAAACAGTTCACGCCATGATGGAAAGGAAGTTGATGAAGGAGCCTGGGAAACTAAAATTAGTCACAGAGAGAAACGACAGCAGCGTAAACGTGATAAGGTGCTGACTGATTCTGGTTCATTGGATTCAACTATCCCTGGGATAGAAAATACCATCACAGTTACCACCGAGCAACTTACAACCGCATCATTTCCTGTTGGTTCCAAGAAGAATAAAGGTGATTCTCATCTAAATGTTCAAGTTAGCAACTTTAAATCTGGAAAAGGAGATTCTACACTTCAGGTTTCTTCAGGATTGAATGAAAACCTCACTGTCAATGGAGGAGGCTGGAATGAAAAGTCTGTAAAACTCTCCTCACAGATCAGTGCAGGTGAGGAGAAGTGGAACTCCGTTTCACCTGCTTCTGCAGGAAAGAGGAAAACTGAGCCATCTGCCTGGAGTCAAGACACTGGAGATGCTAATACAAATGGAAAAGACTGGGGAAGGAGTTGGAGTGACCGTTCAATATTTTCTGGCATTGGGTCTACTGCTGAGCCAGTTTCTCAGTCTACCACTTCTGATTATCAGTGGGATGTTAGCCGTAATCAACCCTATATCGATGATGAATGGTCTGGGTTAAATGGTCTGTCTTCTGCTGATCCCAACTCTGATTGGAATGCACCAGCAGAAGAGTGGGGCAATTGGGTAGACGAAGAAAGAGCTTCACTTCTAAAGTCCCAGGAACCAATTCCTGATGATCAAAAGGTCTCAGATGATGATAAAGAAAAGGGAGAGGGAGCTCTTCCAACTGGGAAATCCAAAAAGAAAAAAAAGAAAAAGAAGAAGCAAGGTGAAGATAACTCTACTGCACAGGACACAGAAGAATTAGAAAAAGAGATTAGAGAAGACCTTCCAGTGAATACCTCTAAAACCCGTCCAAAACAGGAAAAAGCTTTTTCCTTGAAGACCATAAGCACTAGTGATCCAGCCGAAGTACTCGTCAAAAATAGCCAGCCTATCAAGACTCTTCCACCTGCTACTTCTACCGAGCCATCTGTAATCTTATCAAAAAGTGATTCTGACAAGAGCTCTTCCCAAGTGCCGCCAATACTACAAGAGACAGATAAATCCAAGTCAAATACCAAGCAAAATAGTGTGCCTCCTTCACAGACCAAGTCTGAAACTAGCTGGGAATCTCCCAAACAAATAAAAAAGAAGAAAAAAGCCAGACGAGAAACGACGCGTACGCGGCCGCTCGAGGATTATAAGGATGACGACGATAAATTCGTCGAGCACCACCACCACCACCACTAATAAGGTTTATCCGATCCACCGGATCTAGATAAGATATCCGATCCACCGGATCG |

MTDH, Metadherin; α-SMA, alpha-smooth muscle actin.

| Urodynamic index | Control group | Low dose ketamine group | High dose ketamine group | *P* value |
| --- | --- | --- | --- | --- |
| Urination interval (s) | 111.33 ± 12.05 | 74.24 ± 23.76 | 55 ± 18.53 | 0.01 |
| Unstable contraction frequency | 2.25 ± 0.95 | 4.56 ± 0.45 | 6.75 ± 0.11 | 0.01 |

**Table S2.** The difference of urodynamic indexes between control group and ketamine-associated cystitis rat model group


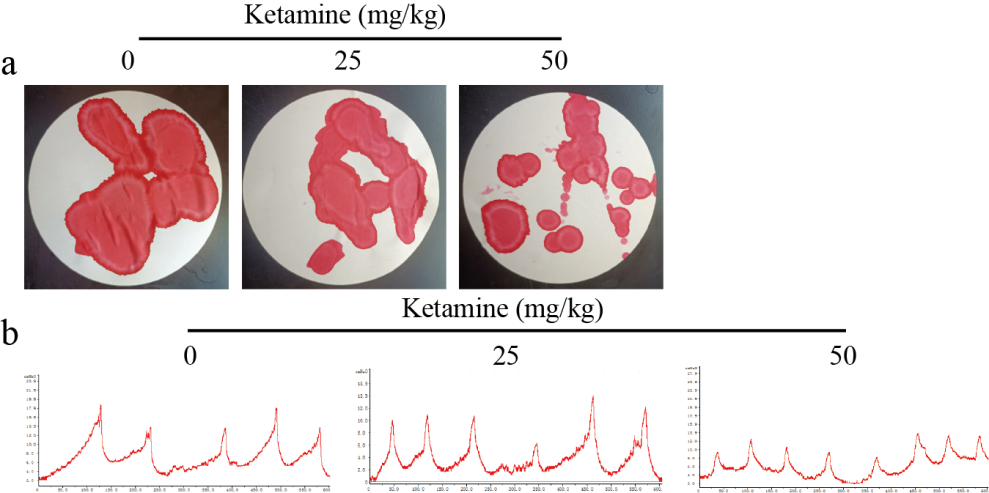


Figure S1. Assessment of bladder function in ketamine model rats. (a) Urination spots in the control and ketamine groups (0mg/kg, 25mg/kg, 50mg/kg). (b) Schematic diagram of the urodynamics of the control group and the ketamine-related cystitis rat model group (0mg/kg, 25mg/kg and 50mg/kg). The abscissa represents time changes, and the ordinate represents pressure changes. N=3.


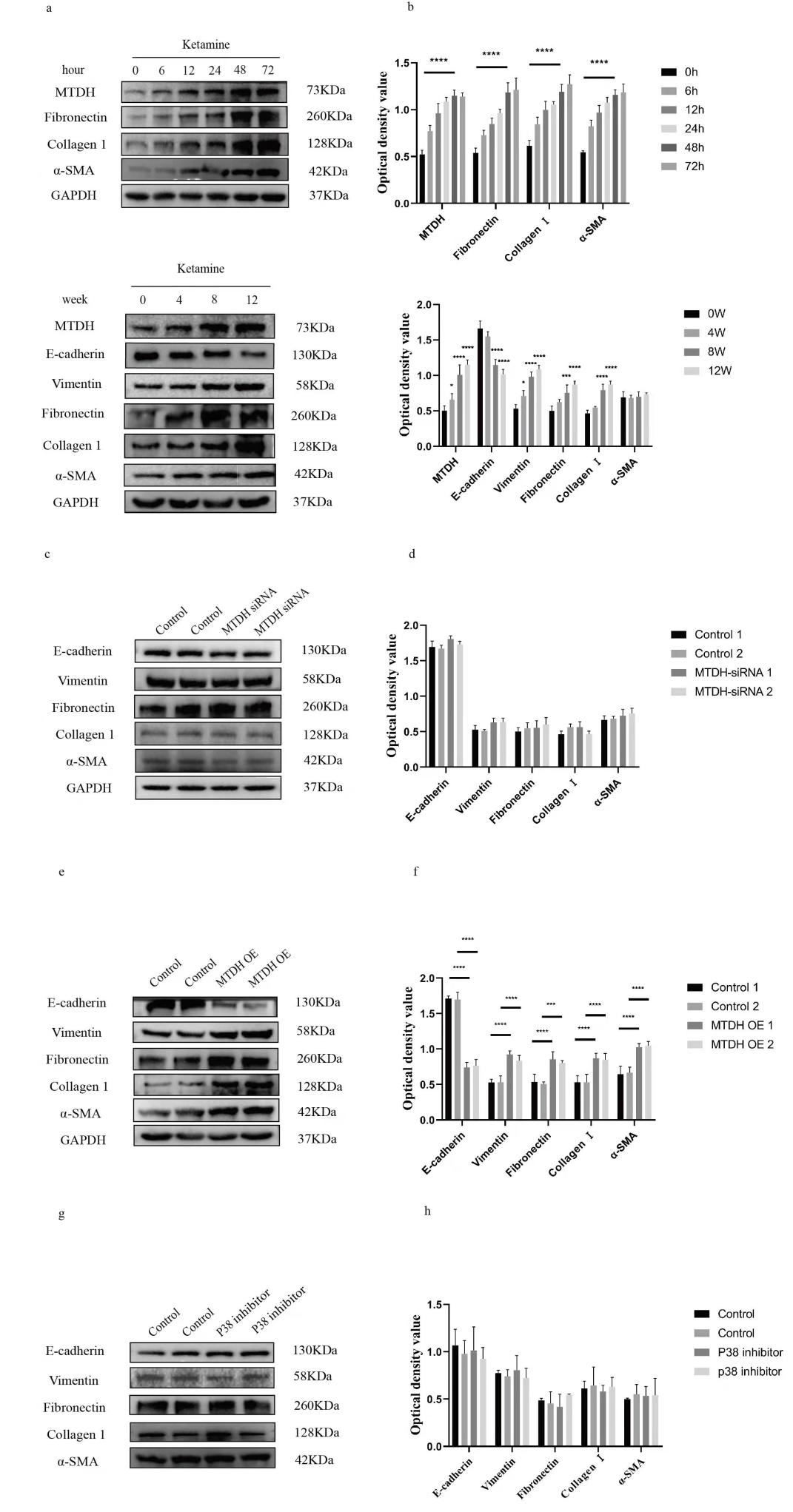


Figure S2. Cell model and animal model time-dependent analysis and siRNA, overexpression and p38 inhibitor group supplementary experiments. (a) Representative bands from Western blot analyses of the levels of the MTDH, vimentin, E-cadherin, fibronectin, collagen Ⅰ, and α-SMA protein in SV-HUC-1 cells and rat bladder after being treated with 1mmol/L and 25mg/kg ketamine, respectively (treated for 0h, 6h, 12h, 24h, 28h, 72h in cells and 0w, 4w, 8w, 12w in rat model). (b) Relative levels of the MTDH, vimentin, E-cadherin, fibronectin, collagen Ⅰ, and α-SMA protein compared to GAPDH. (c) Representative bands from Western blot analyses of the levels of the vimentin, E-cadherin, fibronectin, collagen Ⅰ, and α-SMA protein in SV-HUC-1 cells after being treated with MTDH-siRNA.(d) Relative levels of the vimentin, E-cadherin, fibronectin, collagen Ⅰ, and α-SMA protein compared to GAPDH. (e) Representative bands from Western blot analyses of the levels of the vimentin, E-cadherin, fibronectin, collagen Ⅰ, and α-SMA protein in SV-HUC-1 cells after being treated with MTDH-overecpression plasmid. (f) Relative levels of the vimentin, E-cadherin, fibronectin, collagen Ⅰ, and α-SMA protein compared to GAPDH. (g) Representative bands from Western blot analyses of the levels of the vimentin, E-cadherin, fibronectin, collagen Ⅰ, and α-SMA protein in SV-HUC-1 cells after being treated with P38 inhibitor. (h) Relative levels of the vimentin, E-cadherin, fibronectin, collagen Ⅰ, and α-SMA protein compared to GAPDH. *P < 0.05; ***P < 0.001; ****P< 0.0001. N=3.
